# Translation of the mental health literacy questionnaire for young adults into Chichewa for use in Malawi: preliminary validation and reliability results

**DOI:** 10.1186/s13033-023-00586-7

**Published:** 2023-06-08

**Authors:** Sandra Jumbe, Joel Nyali, Chris Newby

**Affiliations:** 1 Department of Research and Corporate Governance, Millennium University, PO Box 2797, Blantyre, Malawi; 2 School of Medicine, University of Nottingham, Nottingham, UK; 3 Wolfson Institute of Population Health, Centre for Evaluation and Methods, Queen Mary University of London, London, UK

**Keywords:** Mental health literacy, Young people, Questionnaire translation, Survey, Malawi

## Abstract

**Background:**

Mental Health Literacy (MHL) is the ability to recognise mental disorders, have knowledge of professional help available, effective self-help strategies, skills to give support to others, and knowledge of how to prevent mental disorders. Sufficient MHL is linked to better help seeking behaviour and management of mental illness. Assessing MHL importantly helps identify knowledge gaps and inaccurate beliefs about mental health issues, whilst informing development and better evaluation of MHL interventions. This study aimed to translate the English version of a self-reporting Mental Health Literacy questionnaire (MHLq) for young adult populations (16–30 years-old) into Chichewa for use in Malawi and evaluate the psychometric properties of this Chichewa version.

**Methods:**

An established translation methodology was employed, involving back-translation, comparison, forward-translation, comparison, and piloting. The translated Chichewa questionnaire was initially piloted with 14 young adults in a Malawi university, then subsequently administered to 132 young adults in rural community settings across Malawi.

**Results:**

Overall internal consistency of the Chichewa translated MHLq was good (Cronbach’s alpha = 0.67) although subscales’ scores ranged from acceptable (factor 1 and 3) to unacceptable (factor 2 and 4). Confirmatory factor analysis found Factor 1 (Knowledge of mental health problems), Factor 3 (First aid skills and help seeking behaviour) and Factor 4 (Self-help strategies) of the Chichewa version fit very well with related factors of the original English MHLq. For Factor 2 (Erroneous beliefs/stereotypes), 5 out of its 8 items correlated well with the original version. This suggests a four-factor solution is a reasonably good fit to the data.

**Conclusions:**

Use of the Malawian MHLq among Chichewa speaking young adult populations is well supported for factors 1 and 3 but not for factors 2 and 4. More psychometric testing with a larger sample is vital to further validate the questionnaire. Further research is needed to carry out test/re-test reliability statistics.

## Background

Literature in recent years has highlighted a growing concern about the number of youth who are experiencing mental health problems [1, 2]. Preventive approaches are urgently needed to intervene as early as possible to promote positive mental health and well-being mainly because most mental disorders develop during youth [2, 3]. Approximately half of all cases of diagnosed mental disorders in adulthood started by the age of 14 [4]. In Malawi, prevalence rates of common mental disorders (CMDs) like depression and anxiety among youth range from 10 to 30% and this coincides with an existing treatment gap of around 90% in the population [5].

Mental health literacy (MHL) has five basic components [6] which include the ability to recognize mental disorders, along with knowledge of professional help available, effective self-help strategies, skills to give support to others, and knowledge of how to prevent mental disorders. Having sufficient MHL leads to better understanding of risk factors and earlier identification of CMD symptoms, resulting in prevention of mental disorders and subsequent reduced burden of disease [7]. Better MHL also reduces stigma attached to mental illness and informs better planning of help-seeking interventions, resulting in better treatment-seeking attitudes and improved adherence to treatment among diagnosed cases [8].

Evaluating MHL is important for a comprehensive understanding of knowledge gaps and erroneous beliefs about mental health issues, risk factors and treatment-seeking attitudes for mental disorders in given populations. Several instruments like vignette-based interviews and Likert scales have been developed to measure MHL [9, 10]. Recently, a self-reporting measure for assessing MHL in young adults (MHLq) was developed that shows promising ability to consistently assess four aspects of the MHL construct, namely knowledge about mental health problems, erroneous beliefs/stereotypes, first-aid skills and help seeking behaviour, and self-help strategies [11]. This measure can be used by mental health practitioners and researchers to design and evaluate MHL promotion programs, and as a screening tool to identify intervention needs of young adult populations in different settings.

### Study aim

There is very little literature about MHL from developing countries like Malawi. Most studies report findings from Western countries or regions [12]. There is no study assessing MHL among young adults in community settings within Malawi. Moreover, since the recent MHLq for young adults was originally developed in the Portuguese language, translation, back translation, validity testing in local cultures, and psychometric analysis of the data is a pre-requisite before it is used in other languages [11]. The aim of this study was to translate the MHLq into Chichewa for use in the Malawi young adult population, and test the reliability and factor structure of this Chichewa version.

## Methods

### Design

Guidelines by Sousa & Rojjanasrirat [13] for translating, adapting and validating scales for cross cultural use were followed. Steps followed included: (1) two forward translations of the English MHLq version shared by the questionnaire developers [14] into Chichewa, the most commonly spoken local language in Malawi; (2) comparing the translated versions of the target language; (3) blind back translation to the original source language; (4) comparing the blind back-translated version with the original English MHLq and (5) pilot testing of the pre-final Chichewa version with a rural sample of the target population.

In Step 1, ***forward translation*** of the MHLq from English to Chichewa was conducted by two independent bilingual researchers from different backgrounds (SJ - health sciences and psychology; JN - social sciences). Translated drafts were in conversational Chichewa language spoken by Malawians to reflect dialogue relatable to everyday speech, media, newspapers and books.

Step 2 involved ***comparison of forward translations***, where the two bilingual researchers checked and compared their forward translated versions with the original English version and merged them into one Chichewa version. The main change made at this stage was agreement of a consistent translation for ‘mental disorder’ and ‘mental health’ throughout the items. There is no word for ‘schizophrenia’ (items 3 and 27) in Chichewa and this took time to resolve. This preliminary Chichewa version was also reviewed and agreed on by the co-authors and fieldworkers from the National Youth Council of Malawi and Drug Fight Malawi.

In Step 3 (Back translation), one independent bilingual individual with a social science background who was blind to the original English version of the questionnaire back translated the preliminary Chichewa version to English. This translator was Malawian but lived in England therefore had English cultural exposure and knowledge of both English and Chichewa colloquial language.

In Step 4 (Comparison of back translations with the English version), the two bilingual researchers from Step 1 and the Step 3 translator compared the back translated English draft to the original English version. During this process several items were further refined. The panel decided to seek suggestions from local mental health practitioners or advocates to gauge more census around translation of key terms like ‘mental health’, ‘depression’, ‘schizophrenia’ and ‘delusions’. Four contributors provided suggestions on specified key terms which were used to refine several MHLq items e.g., 1, 2, 3, 20, 23. No translation for schizophrenia was identified. However, consensus was that these terms are difficult to translate as there are no existing Chichewa words for them. Therefore, literal translations would do, and fieldworkers could clarify to survey respondents during completion when needed. This process produced a Chichewa version ready for pilot testing. Table 1 represents English and Chichewa versions of the MHLq items.

**Table 1:** MHLq items, MHLq-Young Adult English and Chichewa versions

English items	Chichewa items
Physical exercise contributes to good mental health	Kuchita masewela olimbitsa thupi kumathandiza kuti munthu akhale ndi umoyo wabwino wa ubongo
A person with depression feels very miserable.	Muntha odwala matenda a nkhawa amakhala osakondwa.
People with schizophrenia usually have delusions (e.g., they may believe they are constantly being followed and observed).	Anthu odwala matenda a mubongo ngati ‘schizophrenia’ nthawi zambili amaona zinthu zomwe palibe (mwachitsanzo, atha kukhulupilila kuti wina wake akuwasatila kapena akuwalonda)
If I had a mental disorder I would seek my relatives’ help.	Nditakhala ndi vuto / nthenda ya mubongo, nditha kupempha thandizo kwa achibale
If someone close to me had a mental disorder, I would encourage her/him to look for a psychologist	M’bale wanga kapena nzanga atakhala ndi nthenda ya mubongo, nditha kumulimbikitsa kuti apeze katswiri wa maganizidwe / mubongo
Mental disorders don’t affect people’s behaviours.	Matenda a mubongo sakhuzana ndi makhalidwe a`wanthu
Sleeping well contributes to good mental health.	Kugona mokwanila kumathandiza kuti munthu akhale ndi umoyo wabwino wa ubongo.
If I had a mental disorder, I would seek a psychologist’s help.	Nditakhala ndi nthenda ya mubongo, nditha kufufuza thandizo kwa katswiri wa maganizidwe / mubongo
A person with anxiety disorder may panic in situations that she/he fears.	Munthu odwala matenda a nkhawa, atha kukhala ndi mantha opitilila miyezo zinthu zomwe amaopa zikamachitika
People with mental disorders belong to low-income families.	Anthu omwe ali ndi matenda a mubongo amachokela m’mabanja a ndalama zochepa
If someone close to me had a mental disorder, I would listen to her/him without judging or criticizing.	M’bale wanga kapena nzanga atakhala ndi nthenda ya mubongo, ndingathe kumumvela opanda kumuweluza kapena kumunyogodola
Alcohol use may cause mental disorders.	Kumwa mowa kumatha kuyambitsa matenda a mubongo
Mental disorders don’t affect people’s feelings.	Matenda a mubongo sakhudzana ndi m’mene anthu amamvelela muthupi
The sooner the mental disorders are identified and treated, the better	Matenda a mubongo akadziwika ndi kuchilitsidwa msanga, zinthu zimakhala bwino
Only adults have mental disorders.	Anthu akuluakulu okha ndi omwe amadwala matenda a mubongo
Changes in brain function may lead to the onset of mental disorders	Kusintha kwa magwiridwe antchito a mubongo kutha kuyambitsa nthenda za mubongo
If someone close to me had a mental disorder, I would encourage her/him to see a psychiatrist.	M’bale wanga kapena nzanga atakhala ndi nthenda ya mubongo, nditha kumulimbikitsa kuti akaonane ndi katswiri wa zamisala
If I had a mental disorder I would seek for my friends’ help.	Nditakhala ndi nthenda ya mubongo, nditha kufufuza thandizo kwa anzanga
A balanced diet contributes to good mental health.	Kudya moyenela kumathandizila kuti munthu akhale ndi thanzi labwino mubongo
One of the symptoms of depression is the loss of interest or pleasure in most things.	Chimodzi mwazizindikilo zakukhumudwa ndikusakhala ndi chidwi kapena chisangalalo m’zinthu zambiri
If someone close to me had a mental disorder, I could not be of any assistance	M’bale wanga kapena nzanga atakhala ndi nthenda ya mubongo, sindingathe kumuthandiza.
The symptom’s length is one of the important criteria for the diagnosis of a mental disorder	Nthawi yimene zimatenga kuyeza zizindikilo za matenda a mubongo /misala, ndichinthu chofunika kwambili
Depression is not a true mental disorder.	Matenda okhumudwa si vuto lenileni ya mubongo
Drug addiction may cause mental disorders.	Kuledzela ndi kusuta kwambili kutha kuyambitsa matenda a mubongo
Mental disorders affect people’s thoughts	Matenda a mubongo amakhudza kuganiza kwa anthu
Doing something enjoyable contributes to a good mental health	Kuchita zinthu zosangalatsa kumathandiza kukhala ndi umoyo wabwino wa ubongo
A person with schizophrenia may see and hear things that nobody else sees and hears.	Munthu odwala ‘schizophrenia’ amatha kuwona ndikumva zinthu zomwe wina saziwona kapena kuzimva.
Highly stressful situations may cause mental disorders.	Zinthu zopatsa nkhawa kapena zovuta kwambili zimatha kuyambitsa matenda a mubongo.
If I had a mental disorder, I would seek for a psychiatrist’s help.	Nditakhala ndi matenda a mubongo, nditha kupempha thandizo kwa katswiri wa zamisala

In Step 5 (Pilot testing), the Chichewa questionnaire was piloted with 14 people (university students). In general, all participants said that items of the MHLq were clear i.e., they understood the content of the items.

### Evaluation of the Chichewa MHLq

Participants fulfilling the following criteria were recruited for the purpose of Chichewa MHLq evaluation: young adults living in rural communities; aged 16 to 30 years. Exclusion criteria: not literate in Chichewa; unable to read and write independently. Data collection followed ethical guidelines with all participants providing written informed consent prior to completing the survey. Participants subsequently completed part one of the questionnaire (socio-demographics section), then part two which contained 29 MHLq items. The original English questionnaire that was developed targeting MHL in young people (12–14 years) included 33 items, organised in three subscales: first aid skills and help seeking; knowledge/stereotypes; and self-help strategies [15]. The subsequent Portuguese MHLq for young adults added a fourth factor (xxx), indicating four different areas that make up mental health literacy [14]. These factors and their corresponding item numbers are indicated in Table 2. Surveying was conducted by fieldworkers from Drug Fight Malawi and the National Youth Council of Malawi between 25th March 2021 and 1st May 2021.

Ethics Committee approval was obtained from the National Committee on Research in the Social Sciences and Humanities (NCRSH) on 16th March 2021, prior to commencing with the current study (Ref. No. P12/20/539).

**Table 2:** **Mental health literacy factors quantified in the MHLq.**

Factor	Items (#)
Knowledge of mental health problems	2,3,9,12,16,20,22,24,25,27,28
Erroneous beliefs or stereotypes	6,10,11,13,14,15,21,23
First aid skills and help-seeking behaviour	4,5,8,17,18,29
Self-help strategies	1,7,19,26

### Analysis

To determine sample demographics and characteristics of participants and also levels of missing data, participant characteristics were summarised for the cohort at baseline in a table. To determine internal consistency of the Chichewa translated MHLq, Cronbach’s alpha of all items was calculated. To establish if the same latent structure was visible in the Chichewa translation of MHLq, exploratory factor analysis with varimax rotation was carried out in SPSS. Confirmatory factor analysis was also carried out assuming the four-factor solution as seen in the Portuguese version and allowed for the factors to be correlated using the Lavaan package in R language. Cronbach’s alpha was tested for all items loading on each factor for the confirmatory factor analysis. We used the Kaiser criteria (factors with an eigenvalue greater than 1 to select factors) in the exploratory factor analysis. A factor loading of greater than 0.4 was used to indicate an item is substantially loading on to a factor in both the confirmatory and exploratory factor analysis. Factors were only explored if they satisfied the Kaiser criteria and had three or more items that had a factor loading of 0.4 or greater associated with them. Otherwise, these just indicated a modest bivariate correlation.

To determine convergent validity of the Chichewa version of MHLq we compared means using ANOVA and t tests across the categories of Education and across the baseline question “Do you know anyone who has or had a mental health problem?”. Means and SD with their p-values were summarised. As the questionnaire was only carried out once we could not carry out test/re-test reliability statistics. We documented floor and ceiling effects for each item, to see if the range of the answers to the items were relatable to the Malawi population.

## Results

A total of 132 participants completed the questionnaire from four rural districts in Malawi, namely Kasungu, Mchinji, Mzimba and Salima. Of the young adults, 57% were male, 71% were single, and all participants identified as Malawian. Most participants (57%) were unemployed and a fifth of the sample were in education (n = 27; 21%). Most participants’ (56%) highest educational attainment was predominantly secondary education. 76 participants (58%) reported knowing someone who had a mental health problem. Four people (3%) were not sure if they knew of anyone with such a problem. Regarding the degree of proximity to said person with a mental health problem, most often it was someone not close to the respondent (n = 44; 58%). The other 42% stated that they had a relative or friend with a mental health problem. Table 3 describes demographic characteristics of the survey participants.

**Table 3:** Participants’ characteristics

Participant characteristic	N = 132
Age mean (SD)	22.4 (4.1)
Sex *n(%)*	
Female Male *Missing data*	56 (42) 75 (57) 1
Marital status *n(%)*	
Single Married Divorced *Missing data*	94 (71) 34 (26) 1 (1) 3
Education *n(%)*	
Completed primary education Completed secondary education At college / university Have a bachelor degree *Missing data*	127 42 (32) 74 (56) 8 (6) 3 (2) 5
Occupation *n(%)*	
Employed full time Employed part time Unemployed Student *Missing data*	11 (8) 17 (13) 75 (57) 27 (21) 2
District of residence *n(%)*	
Kasungu Mzimba Salima Mchinji *Missing data*	30 (23) 27 (21) 39 (30) 29 (22) 7
Do you know anyone with a mental health problem? *n(%)*	
Yes No Not sure	76 (58) 52 (39) 4 (3)
Relationship with person (with mental health problem) *n(%)*	
Relative Friend Someone else	11 (14) 21 (28) 44 (58)

### Exploratory factor analysis

105 out of the 132 participants had complete data for all questionnaire items. Using the Kaiser criteria of taking factors with an eigen value greater than 1 we obtained 11 factors accounted for 69% of the variance seen across all items. However only 5 factors had three or more factor loading of greater than 0.4 associated with them. These were factor 1, factor 3, factor 4, factor 6 and factor 8 (see Table 4). Factor 1 had similar items to the ‘First Aid Skills and Help Seeking Behaviour’ factor from the original MHLq. Factors 3, 6 and 8 had similar items to the factor associated with ‘Knowledge of Mental Health Problems’. Factor 4 contained four items that all have different factors associated with them.

**Table 4:** Exploratory factor analysis with factor loading greater than 0.4 shaded

	Factors										
Items	*1	2	*3	*4	5	*6	7	*8	9	10	11
**MHLq4**	0.80	0.01	0.19	-0.12	0.05	0.18	-0.11	-0.16	0.01	-0.01	0.09
**MHLq5**	0.51	0.02	-0.16	0.18	-0.06	0.15	0.54	0.00	-0.08	-0.18	0.07
**MHLq8**	0.79	0.00	-0.08	0.00	0.01	0.07	0.28	0.08	-0.06	0.06	-0.14
**MHLq29**	0.68	0.09	-0.10	0.20	-0.25	0.00	-0.07	0.34	0.09	-0.19	0.02
**MHLq18**	0.80	0.13	0.20	0.01	0.08	-0.12	-0.05	-0.06	-0.10	0.00	0.04
**MHLq19**	0.23	0.18	0.32	0.49	0.26	-0.04	-0.15	-0.14	-0.31	0.09	0.19
**MHLq7**	-0.07	0.11	-0.04	0.66	0.01	0.25	0.20	0.08	0.11	-0.22	-0.20
**MHLq25**	0.05	0.76	0.07	-0.04	0.05	0.04	-0.10	0.18	0.16	0.00	0.07
**MHLq26**	0.10	0.86	0.04	0.06	-0.10	0.00	0.10	0.02	-0.05	-0.04	0.04
**MHLq9**	0.05	0.13	0.56	-0.02	0.21	0.22	0.03	0.17	0.12	-0.16	0.14
**MHLq20**	-0.04	-0.10	0.47	0.00	0.14	-0.02	0.33	0.12	0.65	0.01	0.04
**MHLq14**	0.16	-0.07	0.62	0.07	0.00	0.42	0.19	0.04	-0.09	0.13	0.21
**MHLq17**	0.17	0.21	-0.18	0.66	0.06	0.18	-0.28	0.13	0.09	0.00	0.17
**MHLq22**	-0.07	-0.24	0.15	0.68	0.09	-0.10	0.08	0.03	0.01	0.07	0.03
**MHLq16**	-0.03	-0.19	0.05	0.18	0.78	-0.03	-0.05	0.11	-0.12	0.04	0.17
**MHLq27**	0.01	0.12	-0.10	0.05	0.73	0.11	0.10	0.13	0.11	-0.18	-0.18
**MHLq2**	0.13	0.06	0.31	0.06	0.01	0.75	0.02	0.03	0.08	0.07	0.12
**MHLq12**	0.18	-0.15	-0.17	0.01	0.15	0.42	-0.25	0.49	0.12	-0.01	0.30
**MHLq3**	-0.06	0.03	-0.07	0.09	0.06	0.71	0.12	0.13	-0.10	-0.16	-0.22
**MHLq10r**	0.02	0.00	0.08	-0.02	0.09	0.07	0.82	-0.04	0.09	0.13	0.23
**MHLq28**	-0.13	0.30	0.07	-0.10	0.18	0.14	0.29	0.63	-0.03	-0.07	0.11
**MHLq24**	0.06	0.10	0.20	0.21	0.09	0.05	-0.13	0.83	-0.10	0.12	-0.05
**MHLq6r**	-0.43	0.30	0.10	0.10	0.13	0.14	-0.07	-0.03	0.54	-0.09	0.29
**MHLq13r**	0.00	0.10	-0.18	0.06	-0.14	-0.05	-0.06	-0.14	0.73	0.24	-0.12
**MHLq21r**	-0.02	0.31	-0.04	-0.04	0.36	0.06	0.18	0.04	-0.17	0.54	-0.33
**MHLq23r**	0.04	0.06	-0.02	-0.11	-0.10	-0.06	0.12	-0.11	0.07	0.65	0.42
**MHLq15r**	-0.03	0.13	0.06	0.05	0.01	-0.03	0.26	0.10	-0.05	0.00	0.76
**MHLq1**	-0.04	-0.07	-0.60	-0.04	0.28	0.11	0.10	-0.02	0.05	-0.02	0.17
**MHLq11**	0.11	0.22	-0.02	-0.06	0.15	0.06	0.09	-0.14	-0.21	-0.73	0.12

*factor with three or more factor loading of greater than 0.4

### Confirmatory factor analysis

Confirmatory factor analysis (CFA) was used to determine if the 4-factor solution seen in the Dias and colleagues [14] paper could be formally tested on the data.

Factor loading estimates with std error and p-values are shown in Table 5, items shaded in grey correspond to items that load significantly on to the factor (p < 0.05) onto each factor. Factor 1 (Knowledge of mental health problems) and Factor 3 (First aid skills and help seeking behaviour) of the Chichewa version appeared to fit very well with the original English version of factor 1 and 3. Only item MHLq22 *“The symptom’s length is one of the important criteria for the diagnosis of a mental disorder”*, did not fit into Factor 1 regarding knowledge of mental health problems and item MHLq17 *“If someone close to me had a mental disorder, I would encourage her/him to see a psychiatrist.”* did not fit into Factor 3.

With Factor 2 (Erroneous beliefs/stereotypes) 5 out of the 8 items correlated well with the English version, with loadings over 0.2. However, 3 items namely MHLq 11, 13r and 21r did not fit into the factor structure, suggesting some stereotypes and erroneous beliefs are the same as the English version but not the specific ones below;

*If someone close to me had a mental disorder, I would listen to her/him without judging or criticising (MHLq11)*.

*Mental disorders don’t affect people’s feelings (MHLq13r)*.

*If someone close to me had a mental disorder, I could not be of any assistance (MHLq21r)*.

Factor 4 (Self-help strategies) also fit okay with the original version apart from item MHLq1 *“Physical exercise contributes to good mental health”* which was almost negatively correlated with the factor. So, the belief by most survey respondents was that physical exercise is not a self-help strategy.

**Table 5:** Confirmatory factor analysis of Chichewa MHLq for four factor solution

Factor	MHLq item	Estimate	Std error	P
F1 Knowledge of mental health problems	MHLq2	0.37	0.08	< 0.001
	MHLq3	0.34	0.12	0.003
	MHLq9	0.37	0.08	< 0.001
	MHLq12	0.50	0.15	< 0.001
	MHLq16	0.34	0.13	0.010
	MHLq20	0.30	0.11	0.006
	MHLq22	0.17*	0.15	0.255
	MHLq24	0.41	0.08	< 0.001
	MHLq25	0.37	0.13	0.005
	MHLq27	0.35	0.13	0.006
	MHLq28	0.56	0.11	< 0.001
F2 Erroneous beliefs or stereotypes	MHLq6r	0.15	0.18	0.406
	MHLq10r	0.32	0.11	0.002
	MHLq11	0.11*	0.17	0.522
	MHLq13r	0.27	0.16	0.098
	MHLq14	0.43	0.09	< 0.001
	MHLq15r	0.40	0.12	0.001
	MHLq21r	0.09*	0.13	0.501
	MHLq23r	0.15	0.18	0.407
F3 First aid skills and help seeking behaviour	MHLq4	1.07	0.13	< 0.001
	MHLq5	0.51	0.11	< 0.001
	MHLq8	0.98	0.12	< 0.001
	MHLq17	0.23	0.14	0.100
	MHLq18	1.07	0.13	< 0.001
	MHLq29	0.83	0.14	< 0.001
F4 Self-help strategies	MHLq1	-0.16	0.13	0.206
	MHLq7	0.25	0.12	0.038
	MHLq19	0.52	0.15	0.001
	MHLq26	0.31	0.11	0.006

### Internal consistency

Cronbach’s Alpha values were good for the total score but ranged from acceptable to unacceptable for the subscales: Total Score (29 items) α = 0.67; Factor 1, knowledge of mental health problems (11 items) α = 0.61; Factor 2, erroneous beliefs/stereotypes (eight items) α = 0.20; Factor 3, first aid skills and help seeking behaviour (six items) α = 0.76; Factor 4, self-help strategies (four items) α = 0.15.

Based on the confirmatory analysis results we excluded the items that did not match to the factor structure found in Dias et al. [14]. This resulted in increased internal consistency, with Cronbach’s alpha values for factor 1, knowledge of mental health problems (10 items) α = 0.63; Factor 2, erroneous beliefs/stereotypes (three items) α = 0.51; Factor 3, first aid skills and help seeking behaviour (five items) α = 0.80; Factor 4, self-help strategies (three items) α = 0.34.

### Convergent validity

We tested the sum of all 29 items as well as just the sum of each factor for mean differences across the two variables “Do you know anyone who has or had a mental health problem?” (yes/no) and Education (completed primary education /completed secondary education/ attending college/university, completed bachelor’s degree). All factors and sum of all items were not significantly different across categories but these are likely to be underpowered as some groups had under 5 people in them due to the small sample size. There was a trend which was nearly significant for the sum of all items increasing with education (p = 0.059) (completed primary education mean (SD) = 113 (10) /completed secondary education mean (SD) = 114(11) / attending college/university mean (SD) = 123 (5), completed bachelor’s degree mean (SD) = 120 (11)).

### Floor and ceiling effects

Ceiling effects were high with all the items having at least 20-73% of their data in the topmost category. Floor effects were reasonable with nearly all items having lowest values under 16% of total with only 1 item having 25% lowest scores.

## Discussion

This current study aimed to translate the English version of a previously developed MHL questionnaire [11, 15] into Chichewa for use in the Malawi young adult population. We also aimed to evaluate the psychometric properties of this Chichewa translated version, specifically testing the reliability and factor structure of this Chichewa MHLq.

Demonstrable significant increases in the prevalence of mental health problems throughout the lifespan across the globe mean the probability of most individuals developing a mental health problem is high [3]. Increasing mental health literacy contributes to the promotion of mental health and may play an important role in early identification and intervention when a mental health problem develops. Unfortunately, there is still very little literature about MHL from developing countries like Malawi [12]. There is even less research around how to appropriately assess MHL in developing country contexts, with most studies using tools that have been developed using cohorts from Western countries or regions [10]. Beyond translation into local language, we also need to deeply consider and prioritise psychometric analyses, validation and relevance of such tools to local cultures, as a pre-requisite to ensure we are indeed measuring what is intended [11].

For the CFA outcomes, there was good evidence for Factor 1 (Knowledge of mental health problems) and Factor 3 (First aid skills and help seeking behaviour) of the Chichewa version when comparing with the outcomes from the Portuguese paper [11] but low evidence for Factor 2 (Erroneous beliefs or stereotypes). Overall, three factors out of the four-factor solution were a reasonably good fit to the data. Three out of the eight items in Factor 2 did not fit into the factor structure, suggesting some stereotypes and erroneous beliefs are not the same as the English version, specifically those regarding judgement or criticism of significant others with a mental health problem (MHLq11), the belief that mental disorders do not affect people’s feelings (MHLq13r) and one’s belief in their ability to assist someone close to them if they had a mental disorder (MHLq21r). Moreover, one item in Factor 4 regarding “Physical exercise contributes to good mental health” did not fit well with Factor 4 in the paper with the Portuguese cohort. In fact, this item (MHLq1) was almost negatively correlated with the factor inferring that most survey respondents believe physical exercise is not a self-help strategy for mental health. Again, only one item did not fit well with correlating factors from the Portuguese cohort for both Factor 1 and Factor 3, namely, MHLq22 *“The symptom’s length is one of the important criteria for the diagnosis of a mental disorder”*, and MHLq17 *“If someone close to me had a mental disorder, I would encourage her/him to see a psychiatrist.”*

We suggest reasons for the discrepancies observed in the CFA outcomes. These reasons mainly reflect potential lack of cultural relevance and environmental context of some of the MHLq items. Item 1 of Factor 4 regarding physical exercise is a good example. There is strong evidence for physical exercise as a good health behaviour, especially its benefits for preventing and managing non-communicable diseases like mental illness globally. Despite this existing evidence, physical activity is a largely neglected modality in most mental health care policies and systems in Africa [16]. This lack of awareness seems to have been reflected in the responses of our Malawian rural community sample. Linking physical exercise specifically to mental health, especially as a self-help strategy is typically a Western or urban concept where populations are likely challenged by the issue of sedentary lifestyles. For someone based in rural Malawi where everyday tasks are typically physically strenuous i.e., collecting water from distant rivers, farming with manual tools, physical exercise may not be perceived or experienced in the same way to those typically confined to office desk environments or easy access to transport. The poor fit for item MHLq22 of Factor 1 is expected considering that many people in Malawi do not know much or talk about mental health, especially types of mental illness and details on symptoms presentation [17]. The low loading on Factor 3’s item MHLq17 likely illustrates the very limited mental health workforce in the country. With only four psychiatrists in the whole country, most Malawians rarely hear of or have access to a psychiatrist [18]. As a result, a significant proportion seek help from traditional healers, spiritual advisers or religious leaders instead [18–20].

For Factor 2, the three items’ low loading may be highlighting complexity around country specific and cultural differences in mental health beliefs and stereotypes [21]. Specifically, that there are differences in sources of prejudice and beliefs around aetiology or causes of mental illness between young adults in rural Malawi [20] and Portugal [22, 23] that result in responses to specific items in Factor 2 not correlating between the two MHLq versions. Most research evidence on stigma related to mental illness is from Western countries and there is limited evidence from an African context [17, 24, 25]. More research focusing on beliefs, stereotypes and stigma related to mental illness in Africa should be conducted to better inform content and relevance of existing and future MHL questionnaires. This also highlights a need for validity testing tools that measure mental health stigma in Malawi [26] and Africa more broadly.

CFA outcomes complement findings from our work exploring the cultural applicability of this MHLq to Malawi which will be published in a separate paper. In that study, we conducted a mixed methods exploration of the MHLq responses, comparing the rural survey responses to focus group discussion participants’ views around how accurate the Chichewa translation was and how culturally relevant the MHLq is for the Malawian context. Qualitative interrogation of the quantitative data highlighted a discrepancy in the factors, especially around stereotypes and beliefs.

There were high ceiling effects indicating that in most of the data people reported in the highest category. This may have affected the variability in the data and may explain the scattered results seen in the exploratory factor analysis. Indeed, focus group participants from our exploratory work on the MHLq’s cultural relevance also questioned the high category scoring, particularly from the ‘knowledge’ subscale items. They felt the items do not sufficiently explore whether survey respondents in rural Malawi understood terms like psychiatrist, psychologist, schizophrenia. They also felt that there may have been a bit of guess work by respondents when completing some of the survey items. They consequently suggested adapting the MHLq by adding ‘I don’t know’ to the Likert scale to better or more explicitly capture respondents’ understanding of questionnaire items.

Overall score for internal consistency was good. However, extremes were noted in the Cronbach alpha scores for the subscales with Factor 1 (α = 0.61) and Factor 3 (α = 0.76) scores being acceptable whilst Factors 2 (α = 0.20) and 4 (α = 0.15) were unacceptable. The very low and unacceptable alpha score for the ‘Self-help strategies’ subscale could be explained by the scale’s low number of items coupled with the small sample size [27]. The internal consistency increased when MHLq items that were not aligning with the factor structure were taken out. In the case of Factor 2, the low alpha may reflect the fact that stereotypes and beliefs differ across countries and cultures [21]. This is especially the case in the context of mental health, where public stigma is not only dependent on the overall understanding of mental health and illness, but also based on specific types of mental illness [28]. Subsequent research should be conducted using larger samples in both rural and urban community settings to reassess internal consistency scores of these subscales. Revision of factors within the Chichewa MHLq version should be considered if the subscales’ alpha scores remain low. In terms of convergent validity, the data was nearly there but the sample size was a bit too small to test for education.

### Strengths of the study

This is the first published study in Malawi that has investigated translation of a tool that can assess mental health literacy and validity testing of the data obtained by the MHLq from rural community settings. More generally it is the first study assessing MHL among young adults in these settings. Clear and well-established guidelines for translating and adapting instruments for cross cultural use brought rigour to the translation process [13]. Input from our local stakeholders improved semantics within translated items.

### Study limitations

The participating sample in the evaluation of this Chichewa MHLq were young people from rural settings, which may limit the generalisability of the questionnaire’s properties to an older population or young people from urban settings in Malawi. We targeted rural settings as people living here are predominantly literate in Chichewa compared to English language. Secondly, we only carried out the questionnaire once so we cannot carry out test/re-test reliability statistics. We plan to use Chichewa responses from the larger dataset of the national survey and compare with this cohort to explore reliability of the questionnaire.

## Conclusions

Our evaluation provides moderate support for the use of the Chichewa version of the MHLq for young adults among Chichewa speaking samples. This questionnaire is a useful tool with exclusive focus on specific MHL dimensions enabling a short, valid and reliable self-report assessment of knowledge about mental health problems, erroneous beliefs/stereotypes, first-aid skills and help seeking behaviour, and self-help strategies. More importantly, translating this MHLq into Chichewa and then conducting validity testing on the data obtained from this translated questionnaire has highlighted the need for more work to be done, such as discerning whether the differences in MHLq constructs observed are due to people understanding the content of the items differently to the UK or Portuguese population. The factor analyses infer some of the questionnaire items are perhaps not culturally appropriate for the context of rural Malawi. Overall, having a validated Chichewa MHLq means mental health professionals and researchers in Malawi can have access to a more culturally and linguistically acceptable tool for assessing mental health literacy. This will aid better design and evaluation of relevant interventions, and accurate screening of the mental health needs in young adult populations in different settings.

## Data Availability

The datasets used and/or analysed during the current study are available from the corresponding author on reasonable request.

## List of abbreviations

CFA: Confirmatory factor analysis
CMDs: common mental disorders
MHL: Mental Health Literacy
MHLq: Mental Health Literacy questionnaire
NCRSH: National Committee on Research in the Social Sciences and Humanities
SD: standard deviation
